# Balancing Environmental Footprints and Nutritional Efficiency in University Canteen Menus: A Scenario-Based Life Cycle Assessment of Menu Architecture

**DOI:** 10.3390/foods15173079

**Published:** 2026-08-31

**Authors:** Merve Çapaş, Şükrü Taner Azgın

**Affiliations:** 1 Department of Nutrition and Dietetics, Faculty of Health Sciences, Erciyes University, Melikgazi, 38039 Kayseri, Türkiye; 2 Department of Environmental Engineering, Faculty of Engineering, Erciyes University, Melikgazi, 38039 Kayseri, Türkiye; stazgin@erciyes.edu.tr

**Keywords:** institutional food services, sustainable food systems, Monte Carlo simulation, protein-adjusted carbon footprint, water footprint, greenhouse gas emissions, university food environment

## Abstract

University canteen menus are increasingly evaluated for their environmental impacts, yet sustainability strategies often focus on single-food substitutions rather than on overall meal structure. This study investigated whether environmental improvements are better achieved through main-dish substitution or through redesigning menu architecture while maintaining nutritional efficiency. An LCA-based dataset of university canteen meals was restructured into a fixed four-course architecture, and four representative scenarios were evaluated for energy, protein, carbon, and water footprints, as well as the protein-adjusted carbon footprint. This was followed by a Monte Carlo simulation (10,000 iterations per scenario) using the observed annual menu dataset. Main-dish substitution reduced the carbon footprint by 27.3% (from 2.355 to 1.713 kg CO_2_e) but decreased protein by 24.9% and increased the water footprint by 12.5%. Restructuring the side and complementary dishes instead reduced the carbon footprint by 23.1% and the water footprint by 67.5% while preserving protein content. Monte Carlo simulation confirmed the strongest profile for the restructuring scenario, with the lowest median protein-adjusted carbon footprint (0.069 kg CO_2_e/g protein) and the highest probabilities of outperforming the reference menu in carbon footprint (86.2%), water footprint (76.9%), and protein-adjusted carbon footprint (81.3%). These findings suggest that optimizing menu architecture, not main-dish substitution alone, offers a practical strategy for reducing environmental burden while maintaining nutritional efficiency in institutional food services.

## 1. Introduction

Sustainable nutrition has become an increasingly important issue at the intersection of environmental sustainability, public health, and community well-being. Contemporary dietary patterns are associated not only with the increasing burden of non-communicable diseases, including cardiovascular disease, diabetes, and obesity, but also with substantial environmental pressures arising from food production and consumption [1]. The global food system contributes considerably to anthropogenic greenhouse gas emissions, freshwater use, land-use change, and natural resource depletion [2,3]. Within this system, animal-based foods, particularly ruminant meat and dairy products, generally have higher environmental impacts than plant-based foods, especially in terms of greenhouse gas emissions, land use, and water requirements [3]. Therefore, improving the sustainability of dietary systems represents an important opportunity to reduce environmental burdens while supporting healthier population-level nutrition outcomes [4].

Institutional food services are key settings in which sustainable nutrition principles can be translated into practice. Among these settings, university canteens occupy a particularly important position because they serve large numbers of young adults during a period in which dietary habits, food preferences, and health-related behaviors continue to develop [5]. The university food environment can influence students’ daily food choices through the availability, affordability, composition, and presentation of meals. In this respect, university canteens should be regarded not solely as food service units but also as institutional environments that can support health promotion and sustainable dietary practices among young adults [6]. Because universities often have direct influence over procurement policies, menu planning, and meal production, they are well-positioned to implement food-service strategies that integrate environmental footprint reduction with nutritional quality [7].

From a public health nutrition perspective, however, sustainable menu planning should not be reduced to simply replacing high-impact foods with lower-impact alternatives. Although substituting animal-based dishes with plant-based options can reduce the carbon and water footprints of meals, such changes need to be evaluated alongside their nutritional consequences [8,9]. A menu that achieves lower environmental impact but compromises energy provision, protein contribution, or overall dietary balance may not fully meet the goals of public health-oriented food service planning. This is particularly relevant in university canteens, where meals are expected to provide affordable, accessible, and nutritionally adequate options for a diverse student population. Therefore, the challenge is not only to design menus with lower environmental footprints, but also to ensure that these menus maintain nutritional contribution, particularly in terms of energy and protein provision, and contribute to healthier institutional food environments [10].

Balancing environmental and nutritional objectives requires a more holistic understanding of meal composition. In real-world catering systems, meals are rarely consumed as isolated food items. Instead, they are usually offered as structured combinations of different components, such as a main dish, side dishes, and a complementary dish. This multi-component structure can be conceptualized as a “menu architecture,” referring to how different meal components interact to shape the overall environmental and nutritional profile of a menu. Within this architecture, high-impact components may appear not only in the main dish but also in side and complementary courses, depending on their ingredient composition, portion size, and frequency of occurrence. Therefore, assessing the environmental burden of institutional menus requires attention to the full course structure rather than to single dishes alone [3,11].

The concept of menu architecture is particularly relevant for institutional catering because it allows sustainability to be addressed at the level of the whole meal rather than through isolated substitutions. For example, replacing a high-impact main dish with a lower-impact alternative may reduce carbon emissions and also alter protein content or overall nutritional value. Conversely, restructuring the side and complementary components of a menu may improve environmental performance while preserving the nutritional contribution of the meal. This perspective is important for public health because sustainable food service interventions should avoid creating a trade-off between ecological benefit and dietary adequacy. Instead, they should seek to identify menu designs that are both environmentally efficient and nutritionally robust.

Despite growing interest in sustainable diets and institutional food services, the literature has often focused on individual food items, single-dish comparisons, or main-dish substitutions. Life cycle assessment studies have provided valuable evidence regarding the environmental impacts of different foods and meals, particularly comparisons between meat-based, vegetarian, and vegan alternatives [9,12]. However, less attention has been paid to how the structure of multi-course institutional menus simultaneously influences environmental impacts and nutritional efficiency. This represents an important gap for universities and other large-scale food service providers seeking practical, evidence-based strategies to reduce environmental footprints without compromising the nutritional quality of the meals they serve [13].

To address this gap, the present study evaluates university canteen menus from a menu architecture perspective using life cycle assessment-based data. Specifically, this study aimed to determine whether environmental improvements in university canteen menus are achieved more effectively through main-dish substitution or through restructuring the overall fixed four-course menu architecture, while considering energy, protein, carbon footprint, water footprint, and protein-adjusted carbon efficiency. The analysis focused on a fixed four-course structure consisting of one main dish, two side dishes, and one complementary dish. Representative four-course scenarios and Monte Carlo simulations were used to evaluate both selected menu examples and the distribution of possible menu combinations within the same architectural structures. By integrating environmental footprint analysis with nutritional efficiency, this study provides a menu-level framework for identifying institutional meal structures that reduce environmental burden while preserving nutritional contribution. Its contribution lies in demonstrating that sustainable university canteen menus should not only be lower in environmental impact but should also maintain nutritional efficiency through carefully designed meal structures.

## 2. Materials and Methods

### 2.1. Study Scope and Menu Architecture Approach

This study was designed as a scenario-based life cycle assessment analysis to evaluate the effects of restructuring menu composition on nutritional outputs and environmental footprint indicators while maintaining a fixed number of courses in university canteen menus. The analytical focus was not to redefine the environmental impacts of individual dishes, but to assess the role of multi-course menu architecture in sustainable menu design using existing recipe-level environmental footprint outputs.

In this respect, the present study addresses a research question distinct from a previous environmental footprint analysis based on the same institutional canteen data. In the previous study, the recipe-level nutritional and environmental profiles of university canteen menus were reported [14]. In the present study, these recipe-level LCA outputs were restructured within a fixed four-course menu architecture, and the effects of main-dish substitution and side/complementary dish restructuring were evaluated through representative scenarios and Monte Carlo simulation.

The analysis consisted of three main components. First, menu items were reclassified according to their position within the menu architecture and dish category, and carbon footprint contribution profiles were calculated. Second, four representative four-course menu scenarios were constructed while maintaining the same number of courses, and these scenarios were compared in terms of nutritional and environmental indicators. Third, a Monte Carlo simulation was applied to evaluate the distribution of possible menu combinations within the same scenario structures.

### 2.2. Recipe-Level Life Cycle Assessment Data

The analytical database used in this study was derived from nutritional composition and environmental footprint outputs of standardized recipes served in a university canteen. For each dish, energy, carbohydrate, protein, fat, carbon footprint, and water footprint values were evaluated at the portion level. Energy was expressed as kcal per portion, macronutrients as g per portion, carbon footprint as kg CO_2_-equivalent per portion, and water footprint as m^3^ per portion.

Carbon and water footprint values were not based on the direct application of generic literature-based coefficients at the menu level. Instead, ingredient quantities in standardized recipes were used as the basis for modeling, and each ingredient was modeled separately using SimaPro version 9.0 (PRé Sustainability, Amersfoort, the Netherlands). This approach allowed the environmental impacts of dishes to be calculated based on recipe composition and portion size rather than on broad food-group coefficients.

The system boundary was defined from raw material production to the kitchen stage. Within this boundary, production stages of food ingredients were included, whereas post-consumption waste management and plate-waste processes were excluded from the analysis. Greenhouse gas emissions were expressed as as kilograms of carbon dioxide equivalent per portion (kg CO_2_-eq/portion) for carbon footprint calculations, while water use per portion was considered for water footprint calculations. Nutritional composition was calculated based on ingredient quantities in standardized recipes and was matched with environmental footprint outputs using the same portion unit.

Prior to simulation analyses, the dataset was screened for unit-format inconsistencies in water footprint values. Values exceeding the expected portion-level range due to decimal-format inconsistencies were corrected according to the dataset-specific decimal scaling. The corrected portion-level values were then used in the scenario and Monte Carlo analyses.

In the present study, dish-level LCA outputs were used as input variables for menu architecture, scenario comparison, and simulation analyses. Repeated occurrences of the same dish across different months or menu days were retained. These repetitions were considered to reflect the observed frequency of each dish within the annual institutional menu pattern and therefore provided a sampling framework based on the observed menu structure, particularly for the simulation stage.

### 2.3. Menu Architecture and Dish Category Definition

Menu items were classified into three structural course groups according to their position in the institutional canteen menu: main dishes, side dishes, and complementary dishes. Main dishes included meat dishes, meatballs, poultry and legume dishes, as well as meat-based vegetable dishes. Side dishes included soups, pasta, pilaf, pastries, and olive oil dishes. Complementary dishes included salads, garnishes, yogurt-based dishes, desserts, compotes, and fruits.

Dishes were further classified at a more detailed dish-category level. The final dish categories included soups, meat dishes, meatballs, poultry dishes, pasta dishes, pilaf dishes, legume dishes, meat-based vegetable dishes, salads, garnishes, pastries, yogurt group, desserts, olive oil dishes, compotes, and fruits. This two-level classification was used for carbon footprint contribution analyses and for defining the dish pools used in the construction of representative scenarios and Monte Carlo simulations.

### 2.4. Calculation of Carbon Footprint Contribution Within Menu Architecture

To determine the contribution of dish categories to the carbon footprint of the annual menu dataset, total carbon footprint values were calculated at the category level. For each category, the total carbon footprint was calculated by summing the carbon footprint values for all dish records in that category. The category contribution percentage was calculated as follows:

Category contribution (%) = category-specific total carbon footprint/total carbon footprint of the menu dataset × 100

In addition, main dishes, side dishes, and complementary dishes were evaluated separately within their own course groups. In this analysis, each course group was treated as 100%, and the carbon footprint contribution of each dish category within that course group was expressed as a within-course percentage share. This approach allowed the carbon burden to be assessed not only by general dish categories but also by the position of each category within the menu architecture.

### 2.5. Construction of Fixed Four-Course Menu Scenarios

A fixed four-course scenario structure was developed to evaluate the effects of changes in menu composition independently of the number of courses. All scenarios consisted of one main dish, two side dishes, and one complementary dish. This structure preserved the multi-course format commonly used in institutional catering while allowing the effects of main-dish substitution and side/complementary dish restructuring to be compared within the same menu architecture.

Scenario 4A was defined as the reference menu structure. This scenario consists of a meatball-based main dish, a soup as the first side dish, a pasta-based side dish as the second side dish, and a dessert as the complementary dish. Scenario 4A was defined as an analytical reference rather than as the mean, median, or modal menu of the observed annual dataset. Its components were selected from dish categories included in the institutional menu dataset to establish a predefined meatball-based four-course configuration consisting of a meatball-based main dish, a soup as the first side dish, a pasta-based side dish as the second side dish, and a dessert as the complementary dish. This configuration provided a stable comparator for isolating two different intervention pathways: main-dish substitution while retaining the remaining menu structure (Scenario 4B), and restructuring the side and complementary components while retaining the main-dish category (Scenario 4C). Scenario 4D represented the combined modification of both elements. Accordingly, percentage changes relative to Scenario 4A should be interpreted as scenario-based contrasts within this predefined comparative framework rather than as changes from the mean or statistically most frequent university canteen menu.

Scenario 4B retained the same side and complementary dish structure as Scenario 4A, but the main dish was changed from the meatball-based group to the legume-based group. This scenario was constructed to isolate the effect of main-dish substitution.

In Scenario 4C, the main dish was retained from the meatball-based group as in Scenario 4A; however, the second side dish was changed from a pasta-based side dish to a cereal-based side dish, and the complementary dish was changed from dessert to salad. This scenario was used to evaluate the effects of restructuring the side and complementary dishes while keeping the main dish unchanged.

In Scenario 4D, the main dish was selected from the legume-based group, the first side dish from soups, the second side dish from cereal-based side dishes, and the complementary dish from the yogurt group. This scenario represented the combined use of a legume-based main dish with a different side and complementary dish structure.

For the representative scenario comparison, one menu combination was selected for each scenario, based on the corresponding four-course structure. Menu-level totals were calculated by summing the portion-level values of the dishes included in each scenario.

### 2.6. Scenario-Level Nutritional and Environmental Indicators

For each representative scenario, total energy, total protein, total carbon footprint, and total water footprint were calculated. Menu-level totals were obtained by summing the portion-level values of the four dishes included in each scenario.

In addition to absolute environmental footprint values, protein-adjusted carbon footprint was calculated to account for the protein contribution of each menu. Carbon footprint per gram of protein was calculated by dividing the total carbon footprint by the total protein content.

Using Scenario 4A as the reference, percentage changes in carbon footprint, water footprint, and protein content were calculated for Scenarios 4B, 4C, and 4D. Thus, both the absolute environmental burden and the direction and magnitude of environmental and nutritional changes relative to the reference menu were evaluated.

### 2.7. Monte Carlo Simulation of Menu Architecture Scenarios

Monte Carlo simulation was performed to avoid limiting the representative scenario analysis to selected menu examples and to evaluate the distribution of possible combinations within the same scenario structures. Each iteration was defined as a complete comparison set containing one simulated menu from each of the four scenarios. Within each scenario, one main dish, one soup, one second side dish, and one complementary dish were independently selected with replacement from the corresponding dish pools.

For Scenario 4A, the selection pools consisted of meatballs, soups, pasta dishes, and desserts. For Scenario 4B, the main-dish pool was changed to legume dishes, while soups, pasta dishes, and desserts were retained. For Scenario 4C, the selection pools consisted of meatballs, soups, pilaf dishes, and salads. For Scenario 4D, legume dishes, soups, pilaf dishes, and yogurt-based dishes were used.

Monte Carlo simulations were conducted in Python version 3.12.13 using the pandas version 2.2.3 and NumPy version 2.3.5 libraries. A single NumPy random-number stream initialized with a fixed seed (2026) was used throughout the simulation to ensure reproducibility. Repeated dish records were retained in the simulation pools; therefore, rather than assuming equal probability for each unique dish, the random selection process provided a frequency-weighted sampling structure in which dishes that appeared more frequently in the observed annual menu dataset had a higher probability of being selected in each iteration. Within every iteration, the scenarios were generated in the fixed order 4A, 4B, 4C, and 4D, and the four course components were sampled in the fixed order of main dish, soup, second side dish, and complementary dish. Although the scenario-specific menus were sampled independently, they were generated jointly within the same pre-specified iteration-level comparison set. Thus, scenario outputs were not generated as separate vectors and subsequently matched by their row indices.

A total of 10,000 simulated menus were generated for each scenario. For each simulated menu combination, total energy, total protein, total carbon footprint, total water footprint, and carbon footprint per gram of protein were calculated. Monte Carlo outputs were summarized by scenario using the median, first and third quartiles (Q1–Q3), mean, standard deviation, and minimum–maximum values. The publication table reported the median and Q1–Q3 because the simulated outputs were used primarily for distributional comparison.

Probability-based comparisons were also performed using Scenario 4A as the reference. For each iteration, the outcome obtained for an alternative scenario was compared with the Scenario 4A outcome generated within the same pre-specified comparison set. For carbon footprint, water footprint, and carbon footprint per gram of protein, a favourable outcome was defined as a value lower than that of Scenario 4A; for protein, it was defined as a value equal to or higher than that of Scenario 4A. The probability estimate was calculated as the number of favourable iteration-level comparisons divided by 10,000. Accordingly, the reported probabilities represent the likelihood that a randomly generated menu from an alternative scenario would outperform an independently generated Scenario 4A menu under the observed frequency-weighted dish distributions.

Uncertainty around the probability estimates was quantified using percentile bootstrap 95% confidence intervals. For each scenario–outcome comparison, an iteration-level binary indicator was defined according to whether the alternative scenario outperformed Scenario 4A. The 10,000 paired binary indicators were resampled with replacement 10,000 times, and the 2.5th and 97.5th percentiles of the resulting bootstrap probability distribution were used as the lower and upper confidence limits, respectively. A separate fixed random seed (2027) was used for the bootstrap procedure.

### 2.8. Statistical and Analytical Approach

Observed recipe-level continuous variables were summarized using mean ± standard deviation and minimum–maximum values where relevant. Monte Carlo outputs were summarized using the median and first and third quartiles (Q1–Q3). Median and Q1–Q3 were selected because the simulated outcomes were generated from mixtures of discrete, frequency-weighted empirical dish pools and were not assumed to follow a normal distribution. Graphical inspection of the simulation outputs indicated varying degrees of asymmetry, particularly for protein and water footprint. Formal normality testing was not used to determine the summary measure because, with 10,000 simulated observations per scenario, such tests may identify minor departures from normality that are not practically meaningful. Mean, standard deviation, and minimum–maximum values were also calculated as supplementary distributional summaries, whereas the publication table reports median and Q1–Q3 to provide a consistent and distribution-independent description across outcomes. Categorical variables were expressed as counts and percentages.

Carbon footprint contribution analyses were performed using category-level sums and percentage contributions. Within-course contribution analyses were performed separately for main dishes, side dishes, and complementary dishes by treating each course group as 100%.

Representative scenario results were reported as absolute values and as percentage changes relative to Scenario 4A. Monte Carlo simulation results were evaluated using distributional summaries and graphical representations. Scenarios were compared in terms of absolute environmental indicators, protein contribution, and protein-adjusted carbon footprint. Probability-based comparisons were calculated as the relative frequency of iteration-level comparisons in which each alternative scenario outperformed Scenario 4A for the corresponding outcome and were reported with percentile bootstrap 95% confidence intervals.

## 3. Results

### 3.1. Dish Category Contributions to Carbon Footprint Within the Menu Architecture

The contribution of dish categories to the total carbon footprint differed markedly across the annual menu dataset (Table 1). The highest contribution was observed for the yogurt group, which accounted for 15.91% of the total carbon footprint, followed by desserts (12.37%), soups (12.04%), meat dishes (9.54%), poultry dishes (9.31%), fruits (7.06%), pasta dishes (6.77%), meatballs (6.36%), and pilaf dishes (5.80%). In contrast, legume dishes, olive oil dishes, and compotes contributed relatively little to the total carbon footprint, with values of 0.43%, 0.48%, and 0.65%, respectively.

The total contribution of each dish category was influenced not only by its mean carbon footprint per portion, but also by its frequency of occurrence in the annual menu. For example, pastries had the highest mean carbon footprint per portion (1.475 ± 0.502 kg CO_2_e/portion), but their overall contribution to the total carbon footprint was 5.22% because of their lower frequency. Similarly, soups had a moderate mean carbon footprint per portion (0.594 ± 0.400 kg CO_2_e/portion), but they contributed 12.04% to the total carbon footprint due to their high occurrence in the menu dataset. This indicates that both per-portion carbon intensity and menu frequency shaped the total carbon burden of each category.

**Figure 1:**
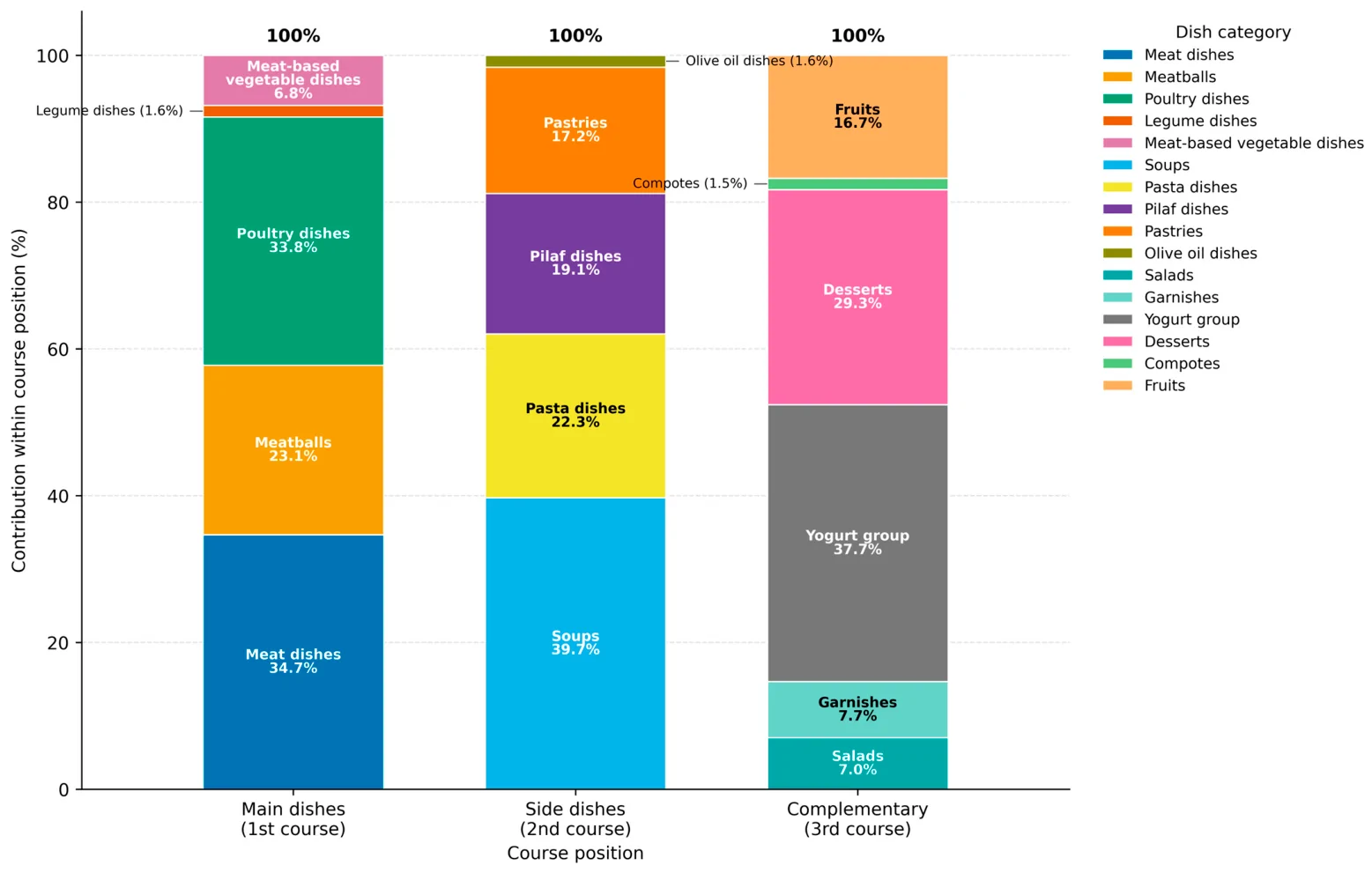
Within-course percentage contribution of dish categories to total carbon footprint by course position. Each course group was treated as 100%, and stacked bars show the relative carbon footprint contribution of dish categories within main dishes, side dishes, and complementary dishes.

When the carbon contribution was examined within the menu architecture, distinct patterns were observed across course positions (Figure 1). Among main dishes, meat and poultry dishes accounted for the largest shares of the carbon footprint, representing 34.7% and 33.8% of the main-dish carbon footprint, respectively. Meatballs contributed 23.1%, whereas meat-based vegetable dishes and legume dishes contributed 6.8% and 1.6%, respectively.

Among side dishes, soups accounted for the largest share of the carbon footprint (39.7%), followed by pasta dishes (22.3%), pilaf dishes (19.1%), pastries (17.2%), and olive oil dishes (1.6%). Within complementary dishes, the yogurt group and desserts were the dominant contributors, accounting for 37.7% and 29.3% of the complementary-dish carbon footprint, respectively. Fruits contributed 16.7%, while garnishes, salads, and compotes contributed 7.7%, 7.0%, and 1.5%, respectively.

Overall, these findings indicate that the carbon footprint of institutional menus was not driven solely by main dishes. Carbon footprint contribution was distributed across main, side, and complementary components, particularly soups, pasta and pilaf dishes, yogurt-based items, and desserts. This supports the relevance of evaluating menu architecture beyond main-dish substitution alone.

### 3.2. Nutritional and Environmental Outcomes of Representative Four-Course Scenarios

The representative four-course scenarios showed distinct nutritional and environmental profiles despite sharing the same menu structure: one main dish, two side dishes, and one complementary dish (Table 2). Scenario 4A, which represented the predefined analytical reference structure composed of tarhana soup, baked meatballs, pasta with cheese, and cocoa pudding, provided 668 kcal and 27.3 g protein. Its total carbon footprint was 2.355 kg CO_2_e, and its water footprint was 0.040 m^3^.

Scenario 4B retained the same side and complementary dishes as Scenario 4A but replaced the meatball-based main dish with chickpea falafel. This scenario had the lowest absolute carbon footprint among the four scenarios (1.713 kg CO_2_e); however, protein decreased to 20.5 g. Its water footprint was 0.045 m^3^, which was higher than that of the reference scenario.

Scenario 4C retained the meatball-based main dish but replaced pasta with cheese with bulgur pilaf and cocoa pudding with seasonal salad. This scenario provided 666 kcal and 27.5 g protein, with a carbon footprint of 1.810 kg CO_2_e and a water footprint of 0.013 m^3^. Thus, compared with Scenario 4A, Scenario 4C combined a lower carbon footprint and markedly lower water footprint while maintaining a similar protein level.

Scenario 4D combined chickpea falafel, tarhana soup, bulgur pilaf, and plain yogurt. This scenario provided the highest energy content among the four scenarios (717 kcal) and included 24.3 g protein. Its carbon footprint was 2.173 kg CO_2_e, and its water footprint was 0.024 m^3^. Although Scenario 4D had a lower water footprint than Scenario 4A, its carbon footprint reduction was smaller than the reductions observed in Scenarios 4B and 4C.

Overall, the representative scenario analysis showed that the lowest absolute carbon footprint was achieved by main-dish substitution in Scenario 4B. However, this reduction occurred together with a lower protein contribution and a higher water footprint. In contrast, Scenario 4C showed a more balanced profile, with reduced carbon and water footprints while preserving protein content.

### 3.3. Relative Changes and Protein-Adjusted Efficiency Across Representative Scenarios

**Figure 2:**
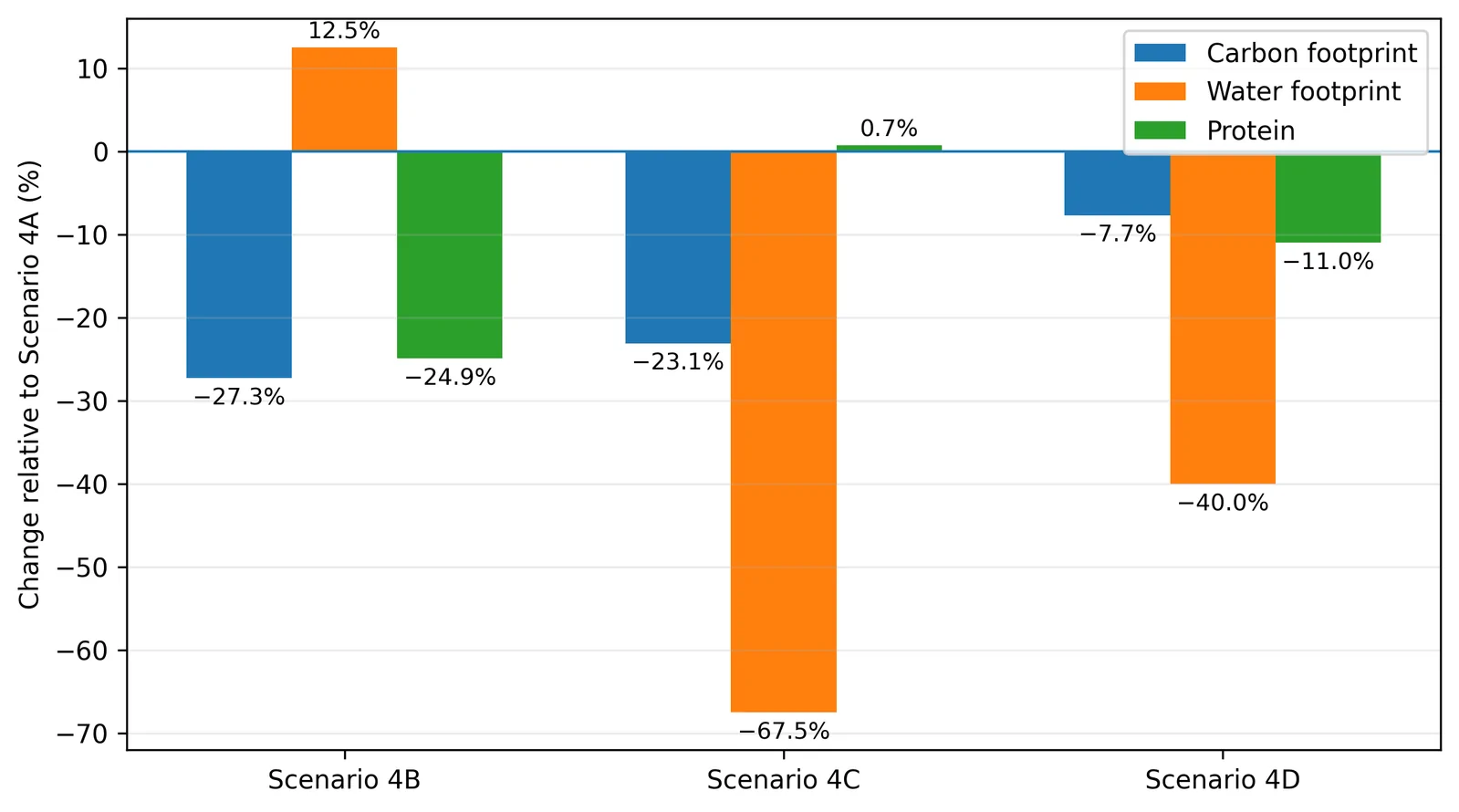
Relative changes in carbon footprint, water footprint, and protein content across representative four-course scenarios compared with the predefined analytical reference, Scenario 4A. Scenario 4A was used as the reference scenario. Negative values indicate reductions, whereas positive values indicate increases relative to Scenario 4A.

Relative changes in environmental and nutritional outcomes were calculated using Scenario 4A as the reference scenario (Figure 2). Scenario 4B showed the greatest reduction in absolute carbon footprint, with a 27.3% decrease compared with Scenario 4A. However, this reduction was accompanied by a 24.9% decrease in protein content and a 12.5% increase in water footprint. Therefore, although replacing the meatball-based main dish with a legume-based main dish reduced the total carbon footprint, this strategy also resulted in a lower protein contribution and did not improve the water footprint.

Scenario 4C showed a 23.1% reduction in carbon footprint and a 67.5% reduction in water footprint compared with Scenario 4A, while protein content remained almost unchanged (+0.7%). This pattern indicates that restructuring the side and complementary components, while retaining the meatball-based main dish, provided a more balanced improvement across environmental and nutritional indicators.

Scenario 4D showed a smaller reduction in carbon footprint than Scenarios 4B and 4C, with a 7.7% decrease compared with Scenario 4A. Its water footprint decreased by 40.0%, but protein content also decreased by 11.0%. Thus, combining a legume-based main dish with bulgur pilaf and plain yogurt improved the water footprint relative to the reference scenario but showed a less favorable carbon and protein-adjusted profile than Scenario 4C.

Protein-adjusted carbon footprint further differentiated the scenarios. Scenario 4A had a CF/protein value of 0.086 kg CO_2_e/g protein, while Scenario 4B had a similar value of 0.084 kg CO_2_e/g protein despite having the lowest absolute carbon footprint. Scenario 4C had the lowest CF/protein value (0.066 kg CO_2_e/g protein), indicating the most favorable carbon efficiency per gram of protein. Scenario 4D had the highest CF/protein value among the alternative scenarios (0.089 kg CO_2_e/g protein). Overall, these findings suggest that absolute carbon footprint reductions should be interpreted together with protein-adjusted indicators, as the lowest-carbon scenario was not necessarily the most protein-efficient option.

### 3.4. Monte Carlo Simulation of Menu Architecture Scenarios

Monte Carlo simulation was performed to evaluate whether the patterns observed in the representative four-course scenarios were maintained when alternative dish combinations were generated within the same predefined menu architectures. A total of 10,000 simulated menus were generated for each scenario using frequency-weighted sampling from the observed annual menu dataset. Each simulated menu preserved the corresponding four-course structure of one main dish, two side dishes, and one complementary dish.

**Figure 3:**
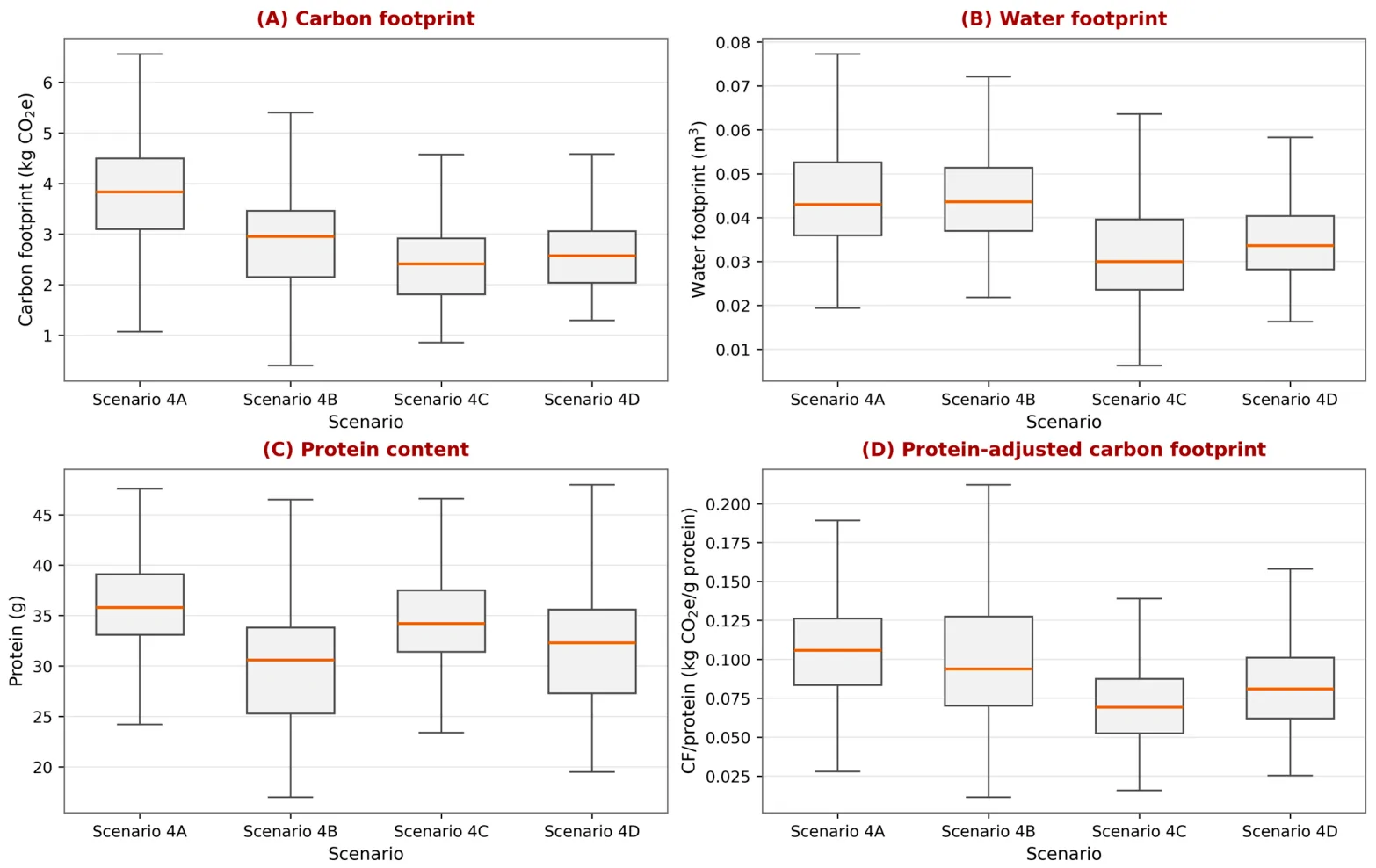
Distribution of nutritional and environmental outcomes across Monte Carlo-simulated four-course menu architecture scenarios. Boxplots represent (**A**) carbon footprint, (**B**) water footprint, (**C**) protein content, and (**D**) protein-adjusted carbon footprint across 10,000 simulated menus per scenario. Boxes indicate the interquartile range, horizontal lines within the boxes indicate the median, and whiskers extend to the most extreme values within 1.5 times the interquartile range. Each scenario preserved its predefined four-course menu architecture, and dishes were selected using frequency-weighted sampling from the observed annual menu dataset.

The simulation results showed distinct distributions across the four menu architecture scenarios (Table 3 and Figure 3). Scenario 4A, representing the reference architecture, had the highest median carbon footprint among the four scenarios, with a median value of 3.834 kg CO_2_e (Q1–Q3: 3.100–4.500). Scenario 4B reduced the median carbon footprint to 2.956 kg CO_2_e (Q1–Q3: 2.156–3.461), but was accompanied by the lowest median protein content among the scenarios, 30.6 g (Q1–Q3: 25.3–33.8), and a median water footprint comparable to that of Scenario 4A.

Scenario 4C showed the most favorable overall profile. Its median carbon footprint was 2.409 kg CO_2_e (Q1–Q3: 1.812–2.917), and its median water footprint was 0.030 m^3^ (Q1–Q3: 0.024–0.040), both lower than those of Scenarios 4A and 4B. At the same time, Scenario 4C maintained a relatively high median protein content of 34.2 g (Q1–Q3: 31.4–37.5), which was closer to Scenario 4A than to Scenario 4B.

Protein-adjusted carbon footprint further supported this pattern. Scenario 4C had the lowest median CF/protein value, 0.069 kg CO_2_e/g protein (Q1–Q3: 0.052–0.088), followed by Scenario 4D, Scenario 4B, and Scenario 4A. Scenario 4D showed an intermediate profile, with a median carbon footprint of 2.571 kg CO_2_e (Q1–Q3: 2.041–3.061), a median water footprint of 0.034 m^3^ (Q1–Q3: 0.028–0.040), and a median CF/protein value of 0.081 kg CO_2_e/g protein (Q1–Q3: 0.062–0.101).

Overall, the Monte Carlo simulation supported the findings obtained from the representative scenario analysis. The results indicated that main-dish substitution alone reduced carbon footprint but was associated with lower protein content, whereas restructuring the side and complementary components produced a more balanced reduction in environmental burden while maintaining a relatively favorable protein contribution.

### 3.5. Probability of Lower Environmental Burden Relative to the Reference Scenario

The probability of achieving a more favorable outcome than the reference architecture was calculated using the pre-specified iteration-level comparisons with Scenario 4A (Table 4). Scenario 4B had a 76.2% probability of producing a lower carbon footprint than Scenario 4A (95% CI: 75.3–77.0%). However, its probability of producing a lower water footprint was 48.5% (95% CI: 47.5–49.5%), and its probability of maintaining protein content equal to or higher than Scenario 4A was 20.0% (95% CI: 19.2–20.8%). The probability of achieving a lower CF/protein value than Scenario 4A was 57.4% (95% CI: 56.5–58.4%).

Scenario 4C showed the strongest overall probability profile. It had an 86.2% probability of producing a lower carbon footprint (95% CI: 85.5–86.8%) and a 76.9% probability of producing a lower water footprint than Scenario 4A (95% CI: 76.0–77.7%). In addition, Scenario 4C had the highest probability of achieving a lower protein-adjusted carbon footprint than the reference scenario, at 81.3% (95% CI: 80.6–82.1%). Although its probability of maintaining protein content equal to or higher than Scenario 4A was 40.5% (95% CI: 39.5–41.5%), this was higher than the corresponding probabilities observed for Scenarios 4B and 4D.

Scenario 4D also showed a high probability of reducing environmental burden, with an 84.7% probability of producing a lower carbon footprint (95% CI: 84.0–85.4%) and a 74.0% probability of producing a lower water footprint than Scenario 4A (95% CI: 73.2–74.9%). Its probability of maintaining protein content equal to or higher than the reference scenario was 28.9% (95% CI: 28.0–29.8%), while its probability of achieving a lower CF/protein value was 71.1% (95% CI: 70.2–72.0%).

Overall, the probability-based comparisons supported the distributional findings. Scenario 4C showed the most consistent combined advantage over the reference scenario for carbon footprint, water footprint, and protein-adjusted carbon footprint, while also showing a higher probability of maintaining protein than the other alternative scenarios.

## 4. Discussion

The present study examined university canteen menus from a menu architecture perspective rather than evaluating sustainability only through individual dish replacement. This distinction is important because institutional meals are not consumed as isolated food items; they are served as structured combinations of main, side, and complementary dishes. The results indicate that the carbon footprint of the annual menu dataset was distributed across different course positions. Although meat-based dishes contributed substantially to the carbon footprint within the main-dish group, side and complementary dishes also represented relevant sources of environmental burden. This pattern suggests that sustainable menu planning in institutional food services should not be limited to replacing the main dish, but should also consider how frequently different components are served and how they interact within the full meal structure.

This interpretation is consistent with the broader food-service LCA literature, which emphasizes that environmental impacts in catering systems are shaped by recipe composition, portion size, production frequency, and menu design rather than by single ingredients alone [7,15,16]. In a university canteen context, Campobasso et al. [11] also approached menu assessment at the food-service level, showing the relevance of evaluating canteen menus through life cycle assessment rather than relying only on general assumptions about food groups. Similarly, Schaubroeck et al. [17] proposed a framework that jointly considers environmental sustainability and nutritional profile in university canteen meals. These studies support the methodological position adopted in the present work: the sustainability of institutional menus is better understood when meals are treated as structured menu systems rather than as isolated products.

The finding that yogurt-based items, desserts, soups, and starchy side dishes contributed meaningfully to the carbon footprint deserves attention. These components are sometimes treated as secondary in sustainable menu discussions because main dishes, especially meat-based dishes, are usually assumed to be the dominant environmental drivers. In the present dataset, however, some side and complementary categories contributed substantially because of their frequency, portion-level footprint, or both. This does not contradict the established evidence that animal-based foods often have higher greenhouse gas emissions than plant-based foods [3,4]. Rather, it indicates that in institutional food services, the total footprint of a menu category depends not only on its intrinsic carbon intensity but also on how often it appears in the menu cycle. For this reason, frequently served items with moderate per-portion footprints may become important contributors at the annual menu level.

The comparison of four-course scenarios further supports the need for a menu-level interpretation. Replacing the meatball-based main dish with a legume-based alternative reduced the absolute carbon footprint, but this change was accompanied by lower protein content and a higher water footprint. This result illustrates a practical trade-off that can be overlooked when sustainability strategies focus only on carbon reduction. Studies comparing meat-based, vegetarian, vegan, and plant-based meals generally report lower greenhouse gas emissions for plant-forward options, but they also emphasize that nutritional outcomes depend on the specific recipe, protein source, portion size, and accompanying dishes [9,13,18]. Therefore, the lower carbon footprint observed after main-dish substitution should not be interpreted as sufficient evidence of a superior menu unless nutritional contribution and other environmental indicators are also considered.

The scenario in which the meatball-based main dish was retained while the side and complementary components were restructured showed a different pattern. In this case, the reduction in carbon footprint was accompanied by a lower water footprint and preservation of protein content. This finding is relevant because it suggests that sustainability improvements may be achieved through strategic changes in menu architecture, even without fully replacing the animal-based main dish. This does not imply that meat reduction is unimportant. Rather, it indicates that in fixed multi-course institutional menus, side dishes and complementary dishes may provide additional intervention points. Evidence from menu optimization and dish-swap studies supports this interpretation. Flynn et al. [19] showed that dish swaps across a weekly menu can support health and sustainability gains, while Grummon et al. [20] reported that simple dietary substitutions may reduce carbon footprints while improving diet quality. In a similar direction, Long et al. [21] suggested that mixed diets can meet nutrient requirements with lower carbon footprints, indicating that sustainability-oriented menu design does not always need to rely on the complete exclusion of animal-based foods.

The protein-adjusted carbon footprint results are central to the interpretation of these scenarios. The lowest absolute carbon footprint was not accompanied by the most favorable carbon efficiency per gram of protein. This distinction is important because institutional meals are expected to provide nutritional value, not only low environmental impact. Moughan [22] emphasized that the choice of protein and sustainability metrics can substantially influence interpretation. Studies using nutrient density or nutrition-adjusted functional units have also shown that foods or diets with lower greenhouse gas emissions are not automatically those with higher nutritional quality [8,10,23,24]. In this context, the protein-adjusted indicator used in the present study helps clarify whether carbon reduction is achieved at the expense of protein contribution. It should be interpreted as a complementary indicator rather than as a replacement for absolute carbon footprint, because protein is only one dimension of nutritional adequacy.

The increase in water footprint observed in the main-dish substitution scenario also indicates that carbon reduction and water reduction may not move in the same direction. This is a relevant issue for sustainable menu planning because many institutional sustainability strategies prioritize greenhouse gas emissions, while water-related impacts may receive less attention. Studies examining carbon–water–food relationships have shown that dietary and food-system changes can involve trade-offs between emissions and water indicators, depending on ingredients, agricultural systems, and regional resource conditions [25,26]. The present findings therefore support a multi-indicator approach in which carbon footprint, water footprint, and nutritional outputs are interpreted together. A menu with a lower carbon footprint may still be less favorable when water footprint or protein contribution is considered.

The Monte Carlo simulation adds an important distributional perspective to the representative scenario comparison. By generating repeated combinations within the same predefined menu structures, the analysis reduced the dependence on a single selected menu example. The simulation results indicated that the scenario based on side- and complementary-dish restructuring maintained a comparatively favorable profile across the carbon footprint, water footprint, and protein-adjusted carbon footprint. This supports the relevance of evaluating scenario structures rather than drawing conclusions from one fixed menu combination. Similar optimization-based studies in school and institutional food services have used modeling approaches to identify menus that balance environmental, nutritional, and practical constraints [27,28,29,30]. The present study contributes to this line of work by applying a frequency-weighted simulation approach to a four-course university canteen menu architecture.

For institutional menu planning, these findings point to the value of evaluating the full meal structure. In university canteens, changing the main dish may be operationally or culturally more visible, but it is not the only possible intervention. Adjusting the second side dish or complementary dish may be more feasible in some settings and may help reduce environmental burden while maintaining a relatively favorable protein contribution. This is particularly relevant where meals are served as fixed combinations and where students expect a certain meal structure. Studies in university and collective catering contexts similarly indicate that environmental assessment should be linked with nutritional quality and food-service feasibility [31,32,33,34]. The implication is not that one universal menu structure should be adopted, but that institutions can use their own recipe and menu-frequency data to identify which components offer the most realistic opportunities for improvement.

### 4.1. Limitations

The study has several limitations that should be considered when interpreting the findings. First, the analysis used recipe-level LCA outputs within a system boundary extending from raw material production to the kitchen stage. Post-consumption processes, actual consumption, plate waste, and waste management were not included. Therefore, the findings represent the modeled environmental and nutritional profiles of the menus rather than their impacts after consumption and waste are considered. Previous studies conducted in school and university food-service settings have shown that food waste and plate waste contribute to environmental impacts and may also result in nutrient losses [35,36,37,38,39]. Second, the nutritional assessment focused on energy and protein, with the protein-adjusted carbon footprint used as an efficiency indicator. Micronutrient adequacy, protein quality, sodium, saturated fat, fiber, and broader diet-quality indices were not evaluated. Third, the scenarios were derived from a single university canteen dataset; therefore, the findings may not be directly generalizable to institutions with different recipes, portion sizes, procurement systems, student preferences, or meal-service models. Fourth, cost, operational feasibility, and consumer acceptability were not assessed. Consequently, the economic feasibility and likely acceptance of the proposed menu changes cannot be inferred from the present findings. Fifth, the bootstrap confidence intervals quantify simulation-based uncertainty conditional on the observed frequency-weighted dish distributions; they do not incorporate uncertainty in the underlying nutritional composition data or life cycle inventory coefficients. Finally, Scenario 4A was defined as an analytical comparator rather than as a statistically derived mean, median, or modal menu. Therefore, the reported percentage changes are conditional on the predefined scenario framework and should not be interpreted as direct estimates of change from the typical menu of the study institution or other university canteens.

### 4.2. Future Directions

Future research should extend the menu-architecture framework in several directions. First, broader system boundaries should incorporate actual consumption, plate waste, and waste-management processes to assess the environmental and nutritional consequences of served meals more comprehensively. Second, nutritional evaluations should include micronutrient adequacy, protein quality, sodium, saturated fat, fiber, nutrient-density scores, and multi-nutrient functional units. Third, multicenter studies involving institutions with different recipes, portion sizes, procurement systems, student populations, and meal-service models are needed to evaluate the generalizability of the findings. Finally, future menu-architecture models should integrate environmental indicators with cost, operational feasibility, and consumer acceptability to support implementation under real-world institutional food-service conditions.

## 5. Conclusions

Rather than treating sustainability as a matter of replacing one dish with another, this study approached university canteen menus as structured meal systems. The results indicate that the environmental profile of a four-course institutional menu is shaped not only by the main dish, but also by the side and complementary components that accompany it. In this context, menu architecture becomes a relevant level of intervention for reducing environmental burden while accounting for nutritional contribution.

The scenario comparisons showed that replacing the meat-based main dish with a plant-based alternative reduced the absolute carbon footprint and also introduced trade-offs in protein content and water footprint. A menu architecture approach allows these trade-offs to be identified more explicitly by considering all courses within the meal. In the present analysis, restructuring the side and complementary components provided a more balanced environmental and nutritional profile than main-dish substitution alone. The simulation analysis further supported the importance of evaluating menu structures rather than relying on a single representative meal.

In practical terms, side- and complementary-dish restructuring in the present analysis meant retaining the meatball-based main dish and soup while replacing the pasta-based second side dish with bulgur pilaf and replacing the dessert with seasonal salad, as represented by Scenario 4C. For institutional menu planners, this finding supports reviewing the full four-course combination rather than focusing exclusively on the main dish. The practical recommendation is to identify higher-impact side and complementary components using institution-specific recipe, menu-frequency, environmental, and nutritional data and to replace them with lower-impact alternatives that maintain an acceptable nutritional contribution. The specific dishes should be adapted to each institution’s menu repertoire, operational conditions, costs, and consumer preferences rather than treating Scenario 4C as a universally applicable fixed menu.

This supports the use of menu-level, multi-indicator assessment as a practical framework for designing university canteen menus that reduce environmental burden while maintaining nutritional efficiency.

## Figures and Tables

**Table 1:** Carbon footprint values and relative contribution of dish categories used for menu architecture analysis.

Dish Category	n	Carbon Footprint, Mean ± SD kg CO_2_e/Portion	Min–Max	Total CF kg CO_2_e	Contribution to Total CF (%)
Soups	166	0.594 ± 0.400	0.124–1.390	98.66	12.04
Meat dishes	69	1.133 ± 0.298	0.420–1.820	78.17	9.54
Meatballs	47	1.108 ± 0.404	0.705–1.800	52.08	6.36
Poultry dishes	83	0.919 ± 0.480	0.030–1.580	76.28	9.31
Pasta dishes	71	0.781 ± 0.274	0.010–1.170	55.48	6.77
Pilaf dishes	96	0.495 ± 0.445	0.010–1.190	47.55	5.80
Legume dishes	23	0.153 ± 0.132	0.040–0.300	3.52	0.43
Meat-based vegetable dishes	22	0.699 ± 0.301	0.180–1.080	15.38	1.88
Salads	103	0.235 ± 0.240	0.010–1.350	24.18	2.95
Garnishes	76	0.348 ± 0.188	0.159–0.690	26.46	3.23
Pastries	29	1.475 ± 0.502	0.928–1.920	42.78	5.22
Yogurt group	97	1.344 ± 0.234	1.120–1.750	130.40	15.91
Desserts	76	1.333 ± 0.694	0.230–2.540	101.30	12.37
Olive oil dishes	14	0.283 ± 0.211	0.109–0.780	3.96	0.48
Compotes	17	0.312 ± 0.287	0.100–0.800	5.30	0.65
Fruits	88	0.658 ± 0.254	0.290–0.920	57.86	7.08

Values are presented as mean ± standard deviation and minimum–maximum. CF: carbon footprint; CO_2_e: carbon dioxide equivalent; SD: standard deviation. Contribution to total CF was calculated as the category-specific total carbon footprint divided by the overall total carbon footprint.

**Table 2:** Nutritional and environmental outcomes of representative four-course menu scenarios.

Scenario	Menu Architecture	Menu Composition	Energy (kcal)	Protein (g)	Carbon Footprint (kg CO_2_e)	Water Footprint (m^3^)	CF/Protein (kg CO_2_e/g Protein)
4A	Main: Meatball-based dish Side 1: Soup Side 2: Pasta-based side dish Complementary: Dessert	Tarhana soup Baked meatballs Pasta with cheese Cocoa pudding	668	27.3	2.355	0.040	0.086
4B	Main: Legume-based dish Side 1: Soup Side 2: Pasta-based side dish Complementary: Dessert	Tarhana soup Chickpea falafel Pasta with cheese Cocoa pudding	696	20.5	1.713	0.045	0.084
4C	Main: Meatball-based dish Side 1: Soup Side 2: Cereal-based side dish Complementary: Salad	Tarhana soup Baked meatballs Bulgur pilaf Seasonal salad	666	27.5	1.810	0.013	0.066
4D	Main: Legume-based dish Side 1: Soup Side 2: Cereal-based side dish Complementary: Dairy-based dish	Tarhana soup Chickpea falafel Bulgur pilaf Plain yogurt	717	24.3	2.173	0.024	0.089

CF: carbon footprint; CO_2_e: carbon dioxide equivalent.

**Table 3:** Monte Carlo simulation results for nutritional and environmental outcomes across four-course menu architecture scenarios.

Scenario	Energy, kcal Median (Q1–Q3)	Protein, g Median (Q1–Q3)	Carbon Footprint, kg CO_2_e Median (Q1–Q3)	Water Footprint, m^3^ Median (Q1–Q3)	CF/Protein, kg CO_2_e/g Protein Median (Q1–Q3)
4A	1116.7 (980.1–1262.1)	35.8 (33.1–39.1)	3.834 (3.100–4.500)	0.043 (0.036–0.053)	0.106 (0.083–0.126)
4B	984.5 (870.7–1130.2)	30.6 (25.3–33.8)	2.956 (2.156–3.461)	0.044 (0.037–0.051)	0.094 (0.070–0.127)
4C	936.2 (855.4–1044.8)	34.2 (31.4–37.5)	2.409 (1.812–2.917)	0.030 (0.024–0.040)	0.069 (0.052–0.088)
4D	831.9 (765.1–909.1)	32.3 (27.3–35.6)	2.571 (2.041–3.061)	0.034 (0.028–0.040)	0.081 (0.062–0.101)

Values are presented as median (Q1–Q3) across 10,000 simulated menus per scenario. Q1: first quartile; Q3: third quartile; CF: carbon footprint; CO_2_e: carbon dioxide equivalent.

**Table 4:** Probability of outperforming Scenario 4A based on iteration-level Monte Carlo comparisons.

Comparison	Lower Carbon Footprint, % (95% CI)	Lower Water Footprint, % (95% CI)	Protein Equal to or Higher, % (95% CI)	Lower CF/Protein, % (95% CI)
4B vs. 4A	76.2 (75.3–77.0)	48.5 (47.5–49.5)	20.0 (19.2–20.8)	57.4 (56.5–58.4)
4C vs. 4A	86.2 (85.5–86.8)	76.9 (76.0–77.7)	40.5 (39.5–41.5)	81.3 (80.6–82.1)
4D vs. 4A	84.7 (84.0–85.4)	74.0 (73.2–74.9)	28.9 (28.0–29.8)	71.1 (70.2–72.0)

Values are presented as probabilities with percentile bootstrap 95% confidence intervals in parentheses. Probabilities were calculated from 10,000 iteration-level comparisons with Scenario 4A. Confidence intervals were obtained from 10,000 bootstrap resamples of the corresponding iteration-level binary comparison indicators. CF: carbon footprint; CI: confidence interval. The intervals quantify simulation-based uncertainty under the specified frequency-weighted sampling model and do not incorporate uncertainty in the underlying nutritional or life cycle inventory coefficients.

## Data Availability

The data presented in this study are available on request from the corresponding author due to restrictions related to the public dissemination of institution-specific standardized recipes and operational food-service information, as well as redistribution restrictions applicable to the underlying inventory data obtained from licensed life cycle assessment resources.

## Abbreviations

The following abbreviations are used in this manuscript:

LCA	Life cycle assessment
CF	Carbon footprint
CO_2_e	Carbon dioxide equivalent
SD	Standard deviation
IQR	Interquartile range
BAP	Scientific Research Projects Coordination Unit
APC	Article processing charge
